# Locoregional recurrence via mucus-mediated extension following lung resection for mucinous tumors

**DOI:** 10.1186/s12885-021-08231-7

**Published:** 2021-04-27

**Authors:** Yo Kawaguchi, Jun Hanaoka, Yasuhiko Ohshio, Keigo Okamoto, Ryosuke Kaku, Kazuki Hayashi, Takuya Shiratori, Akira Akazawa, Mitsuaki Ishida

**Affiliations:** 1 Division of General Thoracic Surgery, Department of Surgery, Shiga University of Medical Science, Tsukinowacho, Seta, Otsu, Shiga 520-2192 Japan; 2 Division of General Thoracic Surgery, Kusatsu General Hospital, Kusatsu, Shiga Japan; 3 Department of Pathology and Laboratory Medicine, Kansai Medical University, Hirakata, Osaka, Japan

**Keywords:** Pulmonary resection, Locoregional recurrence, Mucinous tumor, Mucus extension

## Abstract

**Background:**

Clinically, locoregional recurrences following mucinous tumor resection are often experienced. However, it remains unclear whether mucinous tumors directly affect local recurrence or not, and if so, the mechanism is not known. Therefore, we investigated whether mucinous tumors are associated with locoregional recurrence after pulmonary resection and whether mucus extension is a risk factor for locoregional recurrence.

**Methods:**

The data of 152 patients who underwent pulmonary resection for metastases were reviewed. When mucus was partially or wholly present in the tumor based on macro- or microscopic identification, we assigned the tumor as mucinous. In mucinous tumors, when mucus was identified within the air spaces in the normal lung parenchyma, beyond the edge of the tumor, we assigned the tumor as positive for “mucus extension.”

**Results:**

The 5-year cumulative incidence of locoregional recurrence in patients with mucinous tumors was 48.1%, which was significantly higher than that observed in those with non-mucinous tumors (14.9%). Within the mucinous tumor, the presence of mucus extension beyond the tumor edge was an independent risk factor for locoregional recurrence after pulmonary resection (hazard ratio, 5.52; *P* = 0.019).

**Conclusions:**

During the resection of mucinous cancer, surgeons should maintain sufficient distance from the tumor edge to prevent locoregional recurrences.

## Background

Despite the developments in chemo- and radiotherapy, and treatment with biological agents for patients with pulmonary metastases, surgery remains an important treatment option [1]. Locoregional recurrences following lung resection for metastases are often associated with specific clinicopathological features, such as limited resection, large tumor size, histologic type, and tumor spread through air spaces (STAS) [2–5]. Furthermore, these features are associated with locoregional recurrences and poor prognosis [3, 4]. Mucinous tumors mainly originate from gastrointestinal cancer [6, 7], pseudomyxoma peritonei [8], lung cancer [9], pancreatic cancer [10], uterine cancer [11], ovarian cancer [12], or kidney cancer [13], and sometimes from head and neck cancer [14]. Some mucinous tumors also have poor prognosis following surgery [6, 7, 9, 12].

**Fig. 1:**
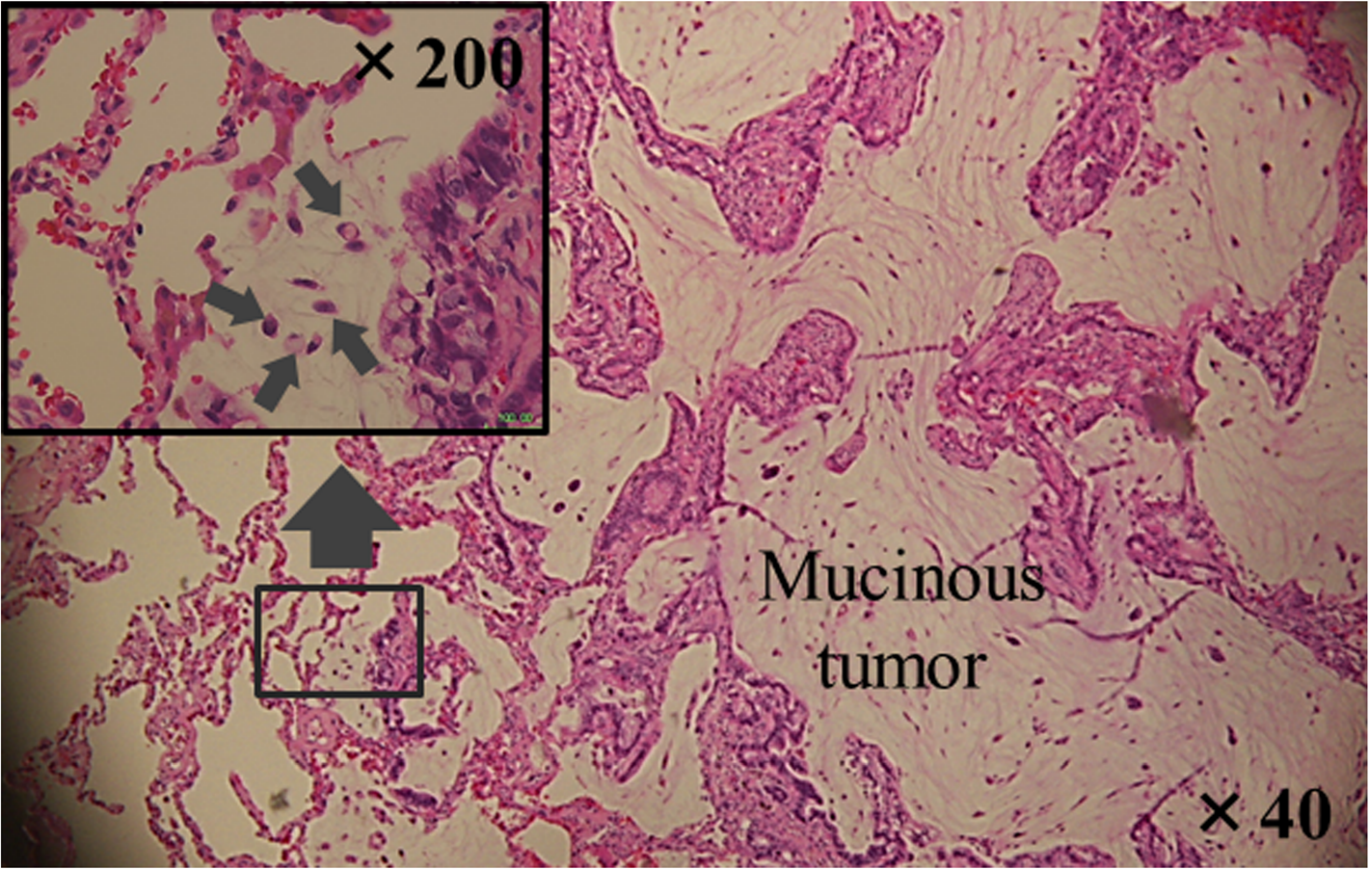
The tumor cells (arrows) exist in the mucus

Clinically, in our hospital, we often observe locoregional recurrences after macroscopic complete resection of mucinous tumors. In addition, we have noticed that mucus derived from the tumor often extends through air spaces into the lung parenchyma adjacent to the tumor edge. We have named this phenomenon “mucus extension.” Mucus extension may be important because tumor cells can spread through mucus-mediated extension [8], as shown in Fig. 1. However, it remains unclear whether this histology could develop tumor recurrence following pulmonary resection or not. Therefore, using the data of a cohort of patients with resected pulmonary metastases, we investigated whether mucinous cancer is associated with locoregional recurrence following pulmonary resection and whether mucus extension is a risk factor for locoregional recurrence according to the surgical procedure type.

## Methods

### Patient cohort

The institutional review board of the Kusatsu General Hospital approved our study (Chairman: Sueyoshi Moritani; Number: 2019–015). The requirement for informed patient consent was waived because of the retrospective nature of the study. All methods were performed in accordance with the journal’s guidelines.

The present study was a retrospective analysis of the medical records of 152 patients who underwent pulmonary resection for metastases at our hospitals between September 2007 and December 2019. The inclusion criteria for patients who underwent surgery were as follows: 1) the primary tumor was controlled and 2) there was no effective therapy other than surgery. We defined the primary tumor as “controlled” when the tumor had been surgically resected and there were no recurrences at the time of pulmonary resection. Furthermore, we enrolled patients whose pulmonary metastases could have been macroscopically resected. The medical record of each patient was reviewed for age, sex, primary tumor, tumor size, number of pulmonary lesions, regions of pulmonary nodules, the surgical procedure for the metastasectomy, and postoperative chemotherapy. We performed wedge resection for metastatic lesions, using an automated suturing device (Endo GIA tri-stapler, Covidien or Endopath stapler Echeron flex, Ethicon, Cincinnati, OH). During the resection, we decided to cut the lung at approximately 1 cm from the tumor edge. We selected segmentectomy, lobectomy, or pneumonectomy when the tumors were located in the hilum or when multiple nodules were present in the same lobe. Lymph node dissection was not routinely performed. Following metastasectomy, the patients were followed-up for a maximum of 5 years. All recurrences were confirmed by radiological assessment. Locoregional recurrence was defined as a nodule occurring on the resection stump of the lung or bronchus and the nodule expanding during follow-up computed tomography examinations.

We classified patients into the mucinous and non-mucinous tumor groups. If mucus was partially or wholly present in the tumor based on macro- or microscopic identification, we assigned the tumor as mucinous. We analyzed the recurrence-free survival and the cumulative incidence of locoregional recurrence in the mucinous tumor group, and compared these values with those of the control (non-mucinous tumor group). If there were multiple lesions in a single patient, we judged that recurrence had occurred in cases where at least one lesion recurred. Furthermore, in the mucinous tumor group, the primary tumor, tumor size, regions of pulmonary nodules, surgical procedure, postoperative chemotherapy, and presence of mucus extension were recorded.

### Histologic evaluation

The surgically resected specimens were fixed by injecting 10% formalin slowly into lung parenchyma by 23-gauge needle. The fixed specimens were cut into 5–10 mm slices with only one stroke using a new knife. All sections that contained a tumor tissue and a surrounding normal lung tissue were embedded in paraffin. Additional 5-μm sections were cut from a selected tissue block and stained with hematoxylin and eosin.

**Fig. 2:**
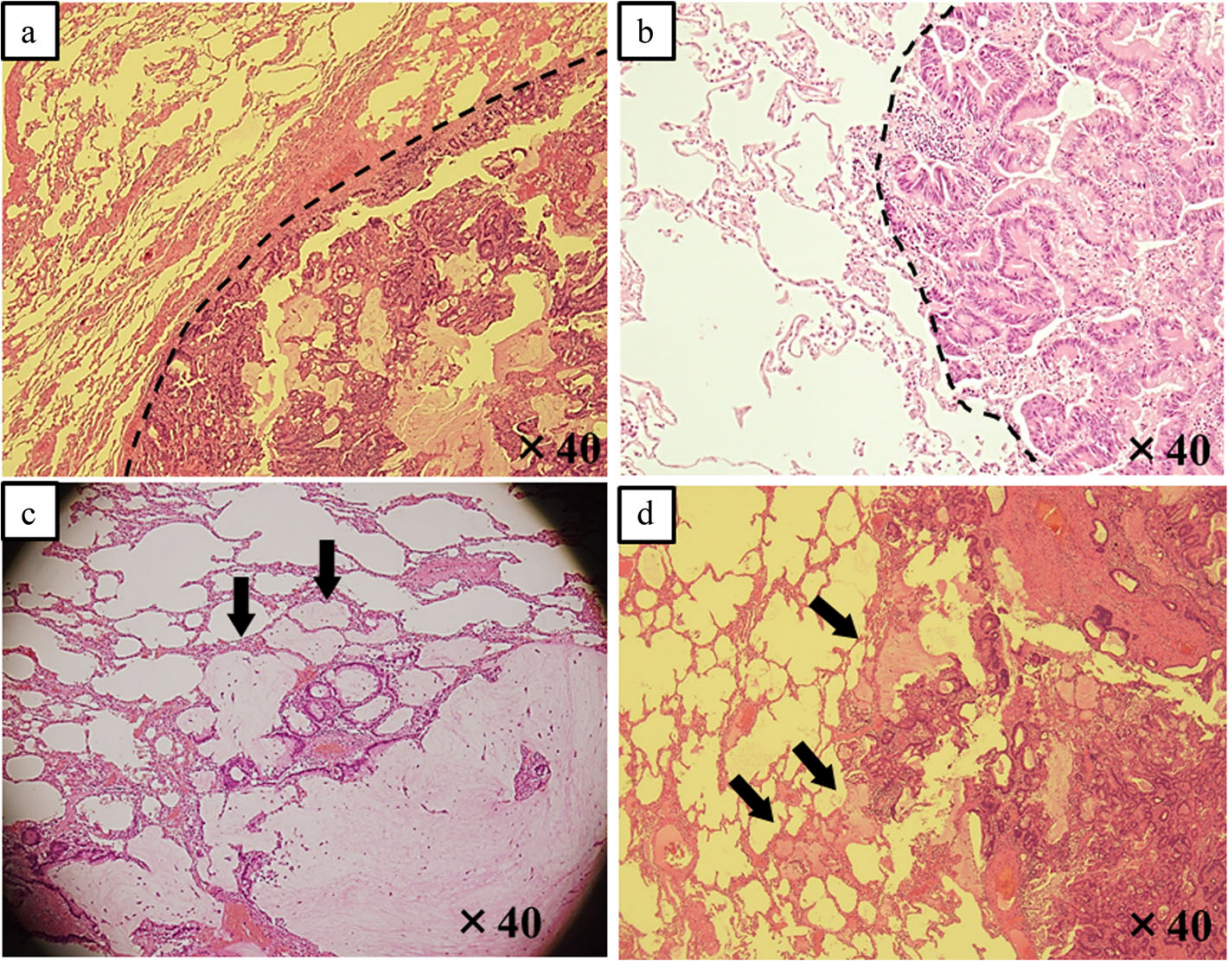
(**a, b**) The border between the tumor/mucus and the normal lung tissue is clear. A border is identified as an easily recognizable smooth surface, indicated by a dotted line. (**c, d**) The border between the tumor/mucus and normal lung tissue is not clear. Mucus is identified within air spaces in the normal lung parenchyma beyond the edge of the tumor (arrows)

We microscopically examined the edge of the tumor or mucus. Specimen sections were divided into two groups: those where the border between the tumor and normal lung tissue was clear (Fig. 2a, b) and those where the border was not clear (Fig. 2c, d). In the former group, the tumor edge was identified as a smooth surface easily recognizable at gross or low-power field examination, as indicated by the dotted line in Fig. 2a and b. In the latter group, we found mucus in the normal alveoli. To distinguish the mucus in the normal alveoli from the mucus in the tumor, we performed the following procedure: First, we observed the size of normal alveoli apart from the tumor cells or mucus. When the mucus existed in the normal size alveoli, we judged the mucus spreading to normal alveoli. When alveoli sizes were expanded or alveolar structure was destroyed by the mucus, we judged it as the mucus within the tumor (indicated by arrows in Fig. 2c, d). We named this phenomenon “mucus extension.” The above pathological diagnoses were performed by YK, and one expert pathologist (MI) checked the diagnoses.

### Statistical analysis

Statistical analysis was performed using SPSS Statistics for Windows, version 25 (IBM Corp., Armonk, NY). Associations between variables were analyzed using Fisher’s exact test (for categorical variables) and the Wilcoxon test (for continuous variables). The Kaplan–Meier method was used to determine the overall survival, relapse-free survival, and cumulative incidence of locoregional recurrence (CIR). The log-rank test was used to compare survival differences for each variable. Cox’s proportional hazards model was used for multivariate analysis. Statistically significant differences were defined as *P* < 0.05.

## Results

### Survival comparison between mucinous and non-mucinous tumors

**Fig. 3:**
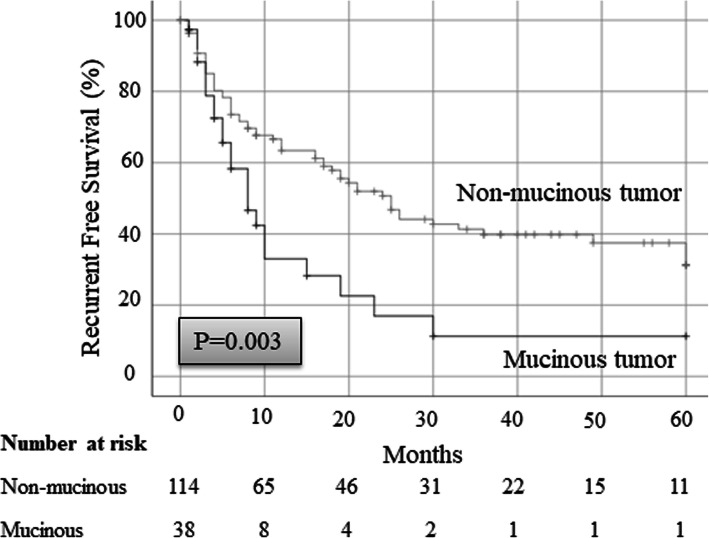
Recurrence-free survival after pulmonary resection in patients with mucinous tumors is 11.3%, which is significantly lower than that observed in patients with non-mucinous tumors (31.2%)

**Fig. 4:**
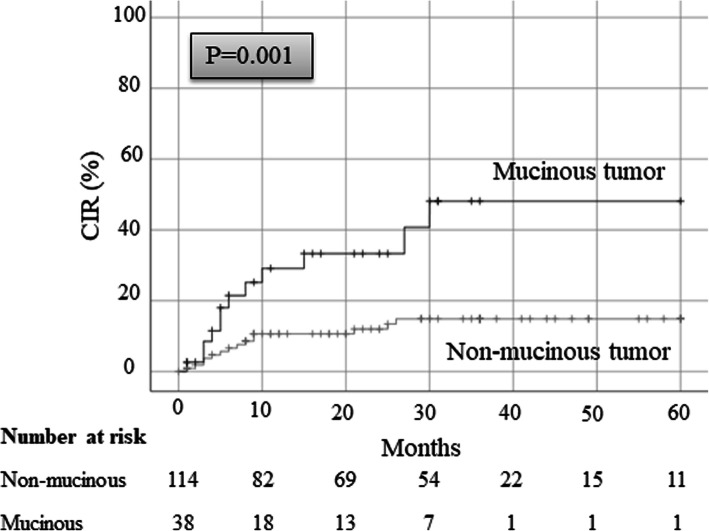
The 5-year CIR rate in patients with mucinous tumors is 48.1%, which is significantly higher than that observed in patients with non-mucinous tumors (14.9%). CIR, cumulative incidence of locoregional recurrence

To examine the association between mucinous tumors and locoregional recurrence, we compared recurrence-free survival between patients with and without mucinous tumors who underwent pulmonary resection for metastases. The characteristics of both groups are presented in Table 1. In total, 38 patients were diagnosed with a mucinous tumor, where the primary tumors were originated from gastrointestinal cancer (26 patients), pseudomyxoma peritonei (10 patients), lung cancer (one patient), and urinary tract cancer (one patient). Moreover, 114 patients were diagnosed with a non-mucinous tumor, where the primary tumors were originated from gastrointestinal cancer (73 patients), kidney/urinary tract cancer (16 patients), head and neck cancer (12 patients), uterine cancer (six patients), lung cancer (one patient), and cancer in other sites (six patients). In gastrointestinal cancers, which were most mucinous tumors, intestinal (Hazard Ratio [HR], 0.98) and pancreatic cancer (HR, 12.0) have a risk of presenting a mucinous tumor (Table 2). The pathological types of tumors, including the number of patients and the number of metastatic lesions, are presented in Table 3. The mucinous tumors comprised adenocarcinoma in 37 patients (97%) and urothelial carcinoma in one patient (3%). The non-mucinous tumors comprised adenocarcinoma, squamous cell carcinoma, clear cell carcinoma, urothelial carcinoma, and other types in 84 (74%), seven (6%), 12 (11%), four (4%), and seven patients (6%), respectively. The recurrence-free survival rate after pulmonary resection in the patients with mucinous tumors was 11.3%, which was significantly lower than that observed in patients with non-mucinous tumors (31.2%; Fig. 3). We suspected that this low recurrence-free survival rate in patients with mucinous tumors was mainly the result of locoregional recurrences. Therefore, we examined the CIR rate and found that the 5-year CIR rate in patients with mucinous tumors was 48.1%, which was significantly higher than that observed in patients with non-mucinous tumors (14.9%; Fig. 4).

**Table 1:** Characteristics of patients with mucinous and non-mucinous tumors

Variables	Mucinous		Non-mucinous		*P* value
	***N*** = 38	%	***N*** = 114	%	
**Age, years**					
Median	61.3	–	64.3	–	0.579
Range	33–77		29–90		
**Gender**					
Male	23	26	65	74	0.450
Female	15	23	49	77	
**Primary tumor**					
Gastrointestinal cancer	26	26	73	74	0.565
Pseudomyxoma peritonei	10	100	0	0	< 0.001
Lung cancer	1	50	1	50	0.355
Kidney, urinary tract cancer	1	6	16	94	0.026
Head and neck cancer	0	0	12	100	0.050
Uterus cancer	0	0	6	100	0.174
The others	0	0	6	100	0.174
**Surgical procedure**					
Partial resection/ enucleation	31	24	97	76	0.271
Segmentectomy	7	29	17	71	0.492
Lobectomy	7	40	9	60	0.224
**Postoperative chemotherapy**					
(+)	16	26	45	74	0.396
(−)	17	20	67	80

**Table 2:** Multivariate analysis of presence or absence of mucus in the gastrointestinal cancers

Variables	Mucinous		Non-mucinous		HR	95% CI	*P* value
	***N*** = 26	%	***N*** = 73	%			
**Gastrointestinal cancer**							
Intestinal cancer	19	22	66	78	0.98	0.43–2.24	0.968
Gastric cancer	0	0	2	100	0.00	–	0.999
Pancreatic cancer	7	78	2	22	12.0	2.20–65.3	0.004
Liver cancer	0	0	3	100	0.00	–	0.999

**Table 3:** The pathological types of tumors, and the number of patients and metastatic lesions

Variables	Mucinous		Non-mucinous	
	Patients	Lesions	Patients	Lesions
**Total**	**38**	**70**	**114**	**179**
**Gastrointestinal cancer**	**26**	**47**	**73**	**112**
adenocarcinoma	26	47	70	105
hepatocellular carcinoma	0	0	3	7
**Pseudomyxoma peritonei**	**10**	**21**	**0**	**0**
adenocarcinoma	10	21	0	0
**Lung cancer**	**1**	**1**	**1**	**1**
adenocarcinoma	1	1	0	0
squamous cell carcinoma	0	0	1	1
**Kidney, urinary tract cancer**	**1**	**1**	**16**	**23**
clear cell carcinoma	0	0	12	18
urothelial carcinoma	1	1	4	5
**Head and neck cancer**	**0**	**0**	**12**	**24**
papillary adenocarcinoma	0	0	8	20
squamous cell carcinoma	0	0	4	4
**Uterus cancer**	**0**	**0**	**6**	**12**
leiomyosarcoma	0	0	3	8
adenocarcinoma	0	0	2	2
squamous cell carcinoma	0	0	1	2
**The others**	**0**	**0**	**6**	**7**
adenocarcinoma	0	0	4	5
adenosquamous cell carcinoma	0	0	1	1
squamous cell carcinoma	0	0	1	1

### Association between locoregional recurrence and mucus extension

We suspected that locoregional recurrence would easily occur in mucinous tumors via mucus-mediated extension. We identified 70 metastatic lesions in 38 patients with mucinous tumors, and 13 lesions developed locoregional recurrences. In contrast, we identified 179 metastatic lesions in 114 patients with non-mucinous tumors, while in 14 lesions, locoregional recurrences were developed. Of the 70 lesions in mucinous tumors, we identified 35 (50%) with mucus extension present. We extracted the potential risk factors of locoregional recurrence as follows: histological type, maximum tumor size, central region of the tumor (tumor existing partially or wholly inside one-third of the area of a pulmonary pleura), postoperative chemotherapy absent, and limited resection (enucleation, wedge resection, or segmentectomy). Associations between these risk factors and mucus extension are analyzed and summarized in Table 4. The risk of developing locoregional recurrence was higher in patients with a maximum tumor size ≥20 mm, tumors in the central region, postoperative chemotherapy absent, and mucus extension. In multivariate analysis, the presence of mucus extension was the independent risk factor for locoregional recurrence (HR, 5.52; *P* = 0.019) (Table 5).

**Table 4:** Clinicopathological associations with locoregional recurrence in mucinous tumors

Variables in mucinous tumor	Lesions	%	3-year CIR (%)	*P* value
**All lesions**	70	–	35.5	–
**Gastrointestinal cancer**	47	67	30.7	0.756
**Pseudomyxoma peritonei**	21	30	Not reached	0.557
**The others**	2	3	Not reached	0.497
**Maximum tumor size**				
< 20 mm	55	79	26.9	0.460
≧20 mm	15	21	57.1	
**Region of the tumor**				
Central	29	41	48.7	0.180
Peripheral	41	59	15.2	
**Surgical procedure**				
Enucleation, wedge, segmentectomy	63	90	40.5	0.156
Lobectomy	7	10	0.0	
**Postoperative chemotherapy**				
(+)	32	62	27.4	0.663
(−)	20	38	42.1	
**Mucus extension**				
(+)	35	50	55.1	0.013
(−)	35	50	8.7

**Table 5:** Multivariate analysis of locoregional recurrence in mucinous tumors

Variables	HR	95% CI	*P* value
**Maximum tumor size≧20 mm**	1.94	0.56–6.77	0.297
**Region of the tumor: Central**	2.01	0.55–57.43	0.293
**Postoperative chemotherapy (−)**	0.81	0.26–2.54	0.717
**Mucus extension: (+)**	5.52	1.37–31.2	0.019

In gastrointestinal mucinous tumors, mucus extension was more frequently observed in pancreatic (HR, 2.54) and intestinal cancers (HR, 0.92) (Table 6).

**Table 6:** Multivariate analysis of mucus extension in gastrointestinal cancers

Mucus extension	(+)		(−)		HR	95% CI	*P* value
	Lesions	%	Lesions	%			
**Gastrointestinal cancer**							
Intestinal cancer	17	46	20	54	0.92	0.33–2.63	0.887
Pancreatic cancer	7	70	3	30	2.54	0.52–12.4	0.247

### Limited resection of mucinous tumors and locoregional recurrence

**Fig. 5:**
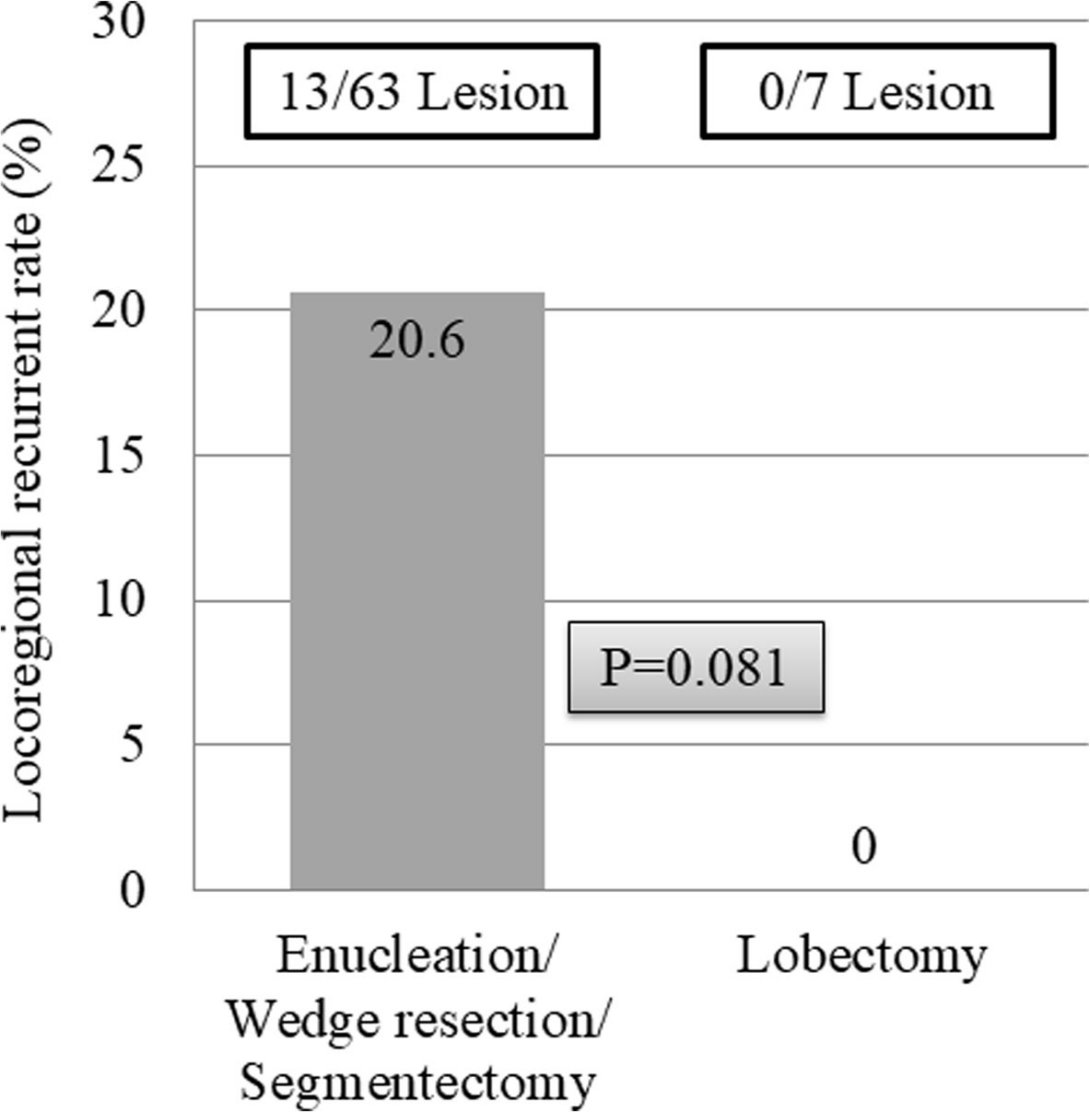
The locoregional recurrence rates were 20.6% for enucleation, wedge resection, or segmentectomy, and 0% for lobectomy

We considered that limited resection may increase the risk of locoregional recurrence. Of 70 lesions, seven and 63 were resected using lobectomy and limited resection (enucleation, wedge resection, and segmentectomy; Table 4), respectively. There was no locoregional recurrence in lesions resected using lobectomy. However, in 13 lesions (20.6%) where limited resection was applied, locoregional recurrence was developed (Fig. 5).

## Discussion

The purpose of this study was to clarify whether mucinous tumors are associated with locoregional recurrence after pulmonary resection and establish whether mucus extension is a risk factor for locoregional recurrence according to the surgical procedure type.

Previously, STAS was not accepted as a form of invasion because it is unique to the lungs. Anatomically, the lungs have air pathways, which permit tumor cells to spread. Kadota et al. identified STAS in 40% of lung adenocarcinoma cases and found that locoregional recurrence after pulmonary resection significantly increased in STAS-positive tumors [2]. Shiono et al. also demonstrated that aerogenous spread with floating cancer cell clusters was an independent prognostic factor [4]; moreover, floating cancer cell clusters and a malignant positive surgical margin in the resected specimens carry significantly higher risk for local recurrence [5] in cases of colorectal pulmonary metastasis. Based on these reports, STAS is coming to be recognized as a pattern of invasion. Previously, we showed that mucinous tumors can spread through mucus-mediated extension [8] in a manner resembling dissemination. Therefore, we hypothesized that the mucus could easily spread through air spaces and that it might be possible for cancer cells to extend via the mucus, resulting in local recurrence. Here, we demonstrated that the recurrence-free survival rate after pulmonary resection in patients with mucinous tumors was significantly lower than that in patients with non-mucinous tumors. This result was similar to those of reports on mucinous tumors found in other parts of the body, which were also associated with high recurrence rates [6, 7, 9, 12].

We considered that mucus extension could be a sensitive marker of locoregional recurrence similar to STAS. We found mucus extension, identified microscopically based on the lack of a distinct border between the tumor and the normal lung tissue, to be present in 50% of mucinous tumors. We considered that mucus extension might cause tumor spread in these cases and, therefore, we hypothesized that mucus extension might be a risk factor for locoregional recurrence. Thus, we extracted the risk factors for locoregional recurrence using the 3-year CIR rate, which identified the factors as follows: maximum tumor size ≥20 mm, tumors in the central region, postoperative chemotherapy absent and mucus extension (univariate analysis). In multivariate analysis, we found that mucus extension was the key independent risk factor for locoregional recurrence following pulmonary resection.

Surgeons need to select the optimal surgical procedure for complete resection of lung tumor. Tumor STAS can be difficult to recognize on the frozen section because STAS tumor cells and alveolar macrophages have similar morphologies. When distinction is difficult, immunohistochemistry for keratin and a macrophage marker, such as CD68, may be needed [2]. Conversely, mucus extension could be identified using a frozen section during the operation. In this study, we showed that locoregional recurrence occurred in patients who underwent enucleation, wedge resection, and segmentectomy, but did not occur in those who underwent lobectomy, suggesting that limited resection may increase the risk of locoregional recurrence. Therefore, in the future, in cases where mucus extension can be identified during the operation, this may help surgeons decide on the need for additional resection or anatomical lung resection. In this study, locoregional recurrence occurred in cases of mucinous tumors with 1-cm resection margins; therefore, margins > 1 cm from the tumor edge should be selected to avoid recurrence.

The main limitation of this study was the heterogeneity of the primary tumor histology. We resected the metastatic tumors irrespective of their primary histology because a recent report showed the effectiveness of pulmonary metastasectomy [15]. However, tumor characteristics, such as growth speed, invasive capacity, and metastatic potential, may differ according to mucus existence and the histology of the primary lesion. In particular, there was histological variability between patients with mucinous and non-mucinous tumors, which might have affected the recurrence-free survival. A second limitation was the small number of cases, which did not provide sufficient power to detect significant differences; therefore, the statistical analysis results may be questionable. Third, we may have underestimated the mucus extension. We observed only the maximum surface of the tumor, and the other surfaces may have potentially contained mucus extension. Fourth, we cannot avoid the possibility of artificial mucus extension. When the lung was resected with automated suturing device or the specimens were injected of formalin, or cut with knife, mucus extension might have been developed artificially by compression. Finally, the diagnosis of local recurrence was equivocal. We confirmed locoregional recurrence through radiological assessment, not by biopsy, which may sometimes misinterpret inflammatory consolidation as locoregional recurrence.

Based on this original study, we intend to spread awareness of the potential risk of postoperative locoregional recurrence in patients with mucinous tumor and mucus extension. Furthermore, a large trial targeting mucinous tumor resection is required to achieve more precise results in the future.

## Conclusions

We demonstrated that mucus extension may be a risk factor for locoregional recurrence after pulmonary resection for lung metastases. Apart from the histology of the primary tumor, whether the tumor is mucinous or non-mucinous should also be considered when pulmonary resection is planned. In the future, we hope that mucus extension can be identified using frozen sections during surgery and that surgeons will consider additional resection to maintain sufficient distance from the tumor edge and prevent locoregional recurrences.

## Data Availability

The data that support the findings of this study are available from the corresponding author upon reasonable request.

## Abbreviations

STAS: Spread through air spaces
CIR: Cumulative incidence of locoregional recurrence
HR: Hazard ratio
CI: Confidence Interval
